# Mutagenicity of an aged gasworks soil during bioslurry treatment

**DOI:** 10.1002/em.20473

**Published:** 2009-03-09

**Authors:** Christine L Lemieux, Krista D Lynes, Paul A White, Staffan Lundstedt, Lars Öberg, Iain B Lambert

**Affiliations:** 1 Mechanistic Studies Division, Chemicals Management DirectorateHealth Canada, Ottawa, Ontario, Canada; 2 Department of Biology, Carleton UniversityOttawa, Ontario, Canada; 3 Department of Chemistry, Umeå UniversityUmeå, Sweden

**Keywords:** bioremediation, polycyclic aromatic hydrocarbons, contaminated soil, mutagenicity

## Abstract

This study investigated changes in the mutagenic activity of organic fractions from soil contaminated with polycyclic aromatic hydrocarbons (PAHs) during pilot-scale bioslurry remediation. Slurry samples were previously analyzed for changes in PAH and polycyclic aromatic compound content, and this study examined the correspondence between the chemical and toxicological metrics. Nonpolar neutral and semipolar aromatic fractions of samples obtained on days 0, 3, 7, 24, and 29 of treatment were assayed for mutagenicity using the Salmonella mutation assay. Most samples elicited a significant positive response on *Salmonella* strains TA98, YG1041, and YG1042 with and without S9 metabolic activation; however, TA100 failed to detect mutagenicity in any sample. Changes in the mutagenic activity of the fractions across treatment time and metabolic activation conditions suggests a pattern of formation and transformation of mutagenic compounds that may include a wide range of PAH derivatives such as aromatic amines, oxygenated PAHs, and S-heterocyclic compounds. The prior chemical analyses documented the formation of oxygenated PAHs during the treatment (e.g., 4-oxapyrene-5-one), and the mutagenicity analyses showed high corresponding activity in the semipolar fraction with and without metabolic activation. However, it could not be verified that these specific compounds were the underlying cause of the observed changes in mutagenic activity. The results highlight the need for concurrent chemical and toxicological profiling of contaminated sites undergoing remediation to ensure elimination of priority contaminants as well as a reduction in toxicological hazard. Moreover, the results imply that remediation efficacy and utility be evaluated using both chemical and toxicological metrics. Environ. Mol. Mutagen. 2009. © 2009 Wiley-Liss, Inc.

## INTRODUCTION

The improper disposal of chemical wastes at industrial sites, such as manufactured gas plants (or gasworks), coking operations, and wood preservation facilities, has resulted in the release of polycyclic aromatic hydrocarbons (PAHs) and other polycyclic aromatic compounds (PACs). The discharged material often takes the form of coal tar or coal-tar creosote, highly complex materials containing hundreds of PAHs and PACs, including a variety of mutagenic carcinogens [WHO, 2004]. Prior to the 1970s, few regulations existed to guide the safe disposal of this type of industrial waste. It was often dumped on or near the site, leaving a legacy of contamination at many sites that have since been abandoned. Soils contaminated with complex PAH mixtures are increasingly recognized as constituting a health and environmental hazard, and their hazardous nature can impede the redevelopment of valuable land for residential, commercial, and/or recreational use.

PAHs and related PACs comprise a class of recalcitrant organic pollutants that are known to be toxic, mutagenic, and carcinogenic to animals and humans [Pickering, 1999]. The assessment of the hazards posed by PAH-contaminated soils ordinarily focuses on a restricted set of priority PAHs, generally 16, that were originally selected by the US Environmental Protection Agency [Keith and Telliard, 1979; US EPA, 2000]. However, a growing body of research now shows that there are many more hazardous and carcinogenic PAHs, and some regulatory agencies (e.g., California EPA and the International Agency for Research on Cancer) now highlight additional compounds (e.g., dibenzo[*a,l*]pyrene and 5-methylchrysene) as probable and possible human carcinogens [IARC, 2008; State of California EPA, 2008]. Moreover, these agencies assert that these compounds should be considered in hazard/risk assessments.

The method commonly employed to monitor the status of a contaminated site (e.g., a PAH contaminated site) that is slated for remediation and redevelopment generally involves chemical-specific surveys for priority chemicals, human health risk assessment for an identified receptor (e.g., construction worker), and reduction of target chemicals to a designated safe level. For example, in Canada, the Canadian Council of Ministers of the Environment (CCME) recommends monitoring a subset of the aforementioned 16 priority PAHs, and calculating the cumulative excess cancer risk associated with the most hazardous PAHs (e.g., benzo[a]pyrene and *dibenz[a,h]* anthracene) [CCME, 2008]. However, many PAH-contaminated sites contain complex mixtures of pollutants, and the chemical profile of all of the toxic compounds in the contaminated soil is rarely, if ever, available. Therefore, it is not known whether the levels of all hazardous compounds at the site decrease concomitantly with the priority PAHs that are monitored during site clean-up.

The removal of PAH-related hazard at contaminated sites is most commonly accomplished via containment (e.g., capping) or incineration. However, these options are costly and do not always produce a site that is ideal for redevelopment. For example, incineration can cost twice as much as bioremediation, and often requires extensive excavation and transportation of contaminated media [Pellerin, 1994]. Bioremediation, a treatment strategy that employs microorganisms, fungi, or plants to convert hazardous organic pollutants into harmless degradation products, is an appealing alternative for the cleanup of PAH-contaminated sites [Wilson and Jones, 1993]. Moreover, bioremediation can leave the site in a state that is suitable for redevelopment and plant cultivation [Wilson and Jones, 1993; Donnelly et al., 2005]. Although bioremediation has been recommended for use at PAH-contaminated sites, its use is not widespread and some researchers have advised caution since the transformation products of PAH degradation that are formed during bioremediation can exhibit increased toxicity, mobility, and bioavailability relative to the parent compounds [Lundstedt et al., 2007].

The transformation of PACs in contaminated soil, through processes such as microbial and fungal degradation, can yield by-products such as hydroxylated and oxygenated PAHs (e.g., oxy-PAHs), and aromatic amines [Cerniglia, 1992; Eriksson et al., 2000; Saponaro et al., 2002]. Several studies have suggested that some products of PAH degradation (particularly oxy-PAHs) may be toxic and/or genotoxic [Donnelly et al., 2005; Lundstedt et al., 2007]. These compounds will therefore contribute to the total risk associated with the contaminated site; however, since their identities are rarely known, their presence, or changes in their concentrations cannot be monitored. Soil genotoxicity assessment using bioassays offers an effective alternative to chemical analyses and can adequately monitor hazard before, during, and after remediation. Toxicity can be used as a measure to assess the efficacy of remediation, and signal the presence of hitherto unidentified compounds that may be of concern. It is therefore important to monitor not only changes in the concentration of noteworthy chemicals (i.e., priority PAHs) at a contaminated site undergoing bioremediation but also changes in toxicological activity.

In an effort to determine the degradation rates of various PAHs and PACs in a heavily contaminated soil from a former gasworks facility, Lundstedt et al. [2003] monitored the levels of many PACs during a pilot-scale bioslurry remediation. They tracked the degradation of over 100 compounds in the soil, including a range of N-, O-, and S-heterocyclics and oxy-PAHs, and showed that the levels of some oxy-PAHs may in fact be increasing over the course of the bioslurry treatment. The authors suggested that these compounds may contribute to toxicological hazard and that measurement of oxy-PAH levels during bioremediation may be warranted. However, reliable assessment of actual changes in the toxicological activity of the complex samples investigated by Lundstedt et al. [2003] requires the use of a bioassay. The Salmonella reverse mutation assay is a logical choice for monitoring changes in the genotoxic activity of PAH-contaminated soils, such as those at former gasworks sites, which are undergoing remediation. Several additional strains are now available (e.g., YG1021, YG1024, YG1041), and these strains provide enhanced sensitivity to several of the aforementioned chemical classes [Watanabe et al., 1989; Hagiwara et al., 1993]. The present study employed the Salmonella reverse mutation assay to monitor changes in the mutagenic activity of the samples examined in the Lundstedt et al. [2003] study. Changes in the mutagenic activity for the nonpolar neutral (i.e., PAHs and alkyl-PAHs) and semipolar aromatic fractions (i.e., N-heterocyclics and oxy-PAHs) were tracked throughout the course of the bioremediation.

## MATERIALS AND METHODS

### Chemicals

All chemicals used for soil extraction and fractionation were analytical grade and obtained from EMD Chemicals (Gibbstown, NJ) unless otherwise specified. Methyl methanesulfonate, 2-nitrofluorene, and 2-aminoan-thracene were obtained from Moltox (Boone, NC). The methodology employed to quantify PAHs in soil extracts was validated using a standard reference material (SRM 2260, National Institute of Standards and Technology, Gaithersburg, MD).

### Soil, Bioslurry Treatment, and Sampling

A 150-kg sample of PAH-contaminated soil was obtained from a former gasworks site at Husarviken in Stockholm, Sweden [Lundstedt et al., 2003]. The gasworks factory on the site was in use from 1893 to 1972. At the time of collection, the site was composed of sandy soil, ash, and demolition debris, all of which is heterogeneously contaminated with coal tar, heavy metals, and cyanide.

The earlier work by Lundstedt et al. provides a thorough description of the bioslurry treatment [Lundstedt et al., 2003]. Briefly, soil from the Husarviken gasworks site was sieved, mixed with water, and the resulting slurry treated in a bioreactor containing a microbial culture designed for the degradation of PAHs in soil (Deutsche Montan Technologie, Essen, Germany), nutrients (Bio-D; Medina Agriculture Products, Hondo, TX), and oxygen (6 mg/l), and was continuously mixed for 29 days. The pH was adjusted to neutral with concentrated sulfuric acid and thus varied from 6.0 to 8.5 throughout the duration of the treatment. One liter aliquots were collected at the beginning of treatment and after 3, 7, 24, and 29 days of treatment. The samples were stored for several weeks at 220 8C until analysis.

### Sample Pretreatment, Extraction, and Fractionation

The pretreatment, extraction, and fractionation of the bioslurry samples was carried out as described in Lundstedt et al [2003]. Briefly, pressurized liquid extraction was used to extract organic compounds from dried samples of remediated soil obtained on days 0, 3, 7, 24, and 29 of the bioremediation treatment. The extracts were applied to 15-mm (inner diameter) open silica gel columns (10% w/w deactivated with water) that were sequentially eluted with 5 ml hexane, 15 ml hexane:dichloromethane (3:1 v/v), and 30 ml dichloromethane. These fractions were reconstituted in 500 μl DMSO (analytical grade, Sigma Aldrich Canada, Oakville, ON, Canada) and stored at 48C until mutagenicity testing with the Salmonella reverse mutation assay. The first fraction contains aliphatic hydrocarbons. The second fraction includes nonpolar neutral compounds such as PAHs, alkyl-PAHs, and S- and O-heterocyclics. The third fraction contains semipolar aromatics including N-heterocyclics and oxy-PAHs. Extensive validation of the extraction and fractionation procedures has been carried out [Lundstedt et al., 2000, 2003, 2006], and analyses of certified reference material showed that recoveries of all PAHs tested except phenanthrene and anthracene were greater than 85%. Acceptable reproducibility has been demonstrated for several PAHs, alkyl-PAHs, oxy-PAHs, and N- and S- heterocyclics (relative standard deviation < 13%) using a standard reference material and a contaminated gasworks soil.

### Salmonella Reverse Mutation Assay

The Salmonella reverse mutation assay was used to assess the muta-genic activity of the same samples examined in the aforementioned Lundstedt et al. [2003] study. Mutagenic activity of the nonpolar neutral and semipolar aromatic fractions of each bioslurry sample (i.e., day 0, 3, 7, 24, and 29) was evaluated using the standard plate incorporation version of the Salmonella reverse mutation assay [Mortelmans and Zeiger, 2000]. The assay was performed according to Mortelmans and Zeiger [2000] with one exception; 0.2 mM histidine and 0.4 mM biotin were added directly to the minimal agar plates rather than the top agar. The first fraction (i.e., aliphatics) was not evaluated for mutagenicity. Earlier analyses on a limited number of soil extracts failed to detect mutagenic activity in this fraction (data not shown). Fractions 2 and 3 (i.e., nonpolar neutral and semipolar aromatic) were tested with and without the addition of a post-mitochondrial supernatant (S9) fraction from Aroclor 1254 induced rat liver (male Sprague-Dawley rats, protein content between 35.7 and 43.5 mg/ml; Moltox, Boone, NC) on *Salmonella* strains TA98, TA100, YG1041, and YG1042. Strains TA98 and YG1041 detect frameshift mutations, whereas strains TA100 and YG1042 detect base pair substitution mutations. YG1041 and YG1042 contain the multicopy pYG233 plasmid, which contains genes encoding O-acetyltransferase (OAT) and the *Salmonella* classic nitroreductase (Cnr) [Hagiwara et al., 1993]. As such, they are highly sensitive to nitroarenes and aromatic amines.

Each fraction of each bioslurry sample was tested at five concentrations (1, 3, 9, 27, and 50 mg equivalent dry soil/plate for the nonpolar neutral fractions and 1, 3, 9, 27, and 81 mg equivalent dry soil/plate for the semipolar aromatic fractions). Each concentration was tested in triplicate, and revertant colonies on each plate were counted using an automated colony counter (Protocol RGB, Model # 9000, Synoptics Ltd, UK).

Positive and negative controls were included in each experiment. When metabolic activation was not used, 2-nitrofluorene was used as a positive control for TA98 (2.5 μg per plate), YG1041 (0.5 μg per plate), and YG1042 (0.5 μg per plate). Methylmethane sulfonate (0.4 μg per plate) was used as a positive control for TA100 without metabolic activation. 2-Aminoanthracene (1 μg per plate) was used as a positive control for all strains with metabolic activation. Negative control samples contained only top agar and bacteria (and S9 where necessary), and did not include DMSO or sample. Previous work in our laboratory has noted identical responses for negative controls that contain DMSO and those that do not (data not shown). All positive and negative controls were tested in triplicate.

### Data Analysis

All statistical analyses were carried out using SAS v. 8.02 for Windows™ (Statistical Analysis Institute, Cary, NC). A sample was classified as mutagenic if it induced a twofold increase in the number of revertants compared to the negative control, and a reproducible concentration-response function was generated in one or more strains. For each mutagenic soil sample, a mutagenic potency (revertants per mg equivalent of dry soil) was calculated using ordinary least squares linear regression on the linear portion of the concentration-response function. All raw data (e.g., plate counts) are readily available from the corresponding author.

## RESULTS AND DISCUSSION

### Transformation of PAHs During Bioremediation

This study tracked changes in the mutagenicity of a PAH-contaminated gasworks soil throughout the course of a pilot-scale bioremediation. Nevertheless, a brief discussion of the changes in the chemical composition of the soil that occurred during the bioremediation is essential for interpretation of the bioassay results. The results presented below constitute an abbreviated summary of the chemical changes noted in the aforementioned Lundstedt et al. [2003] study. For a more thorough description and discussion of the changes in the chemical profiles of the treated soil, the reader is referred to the original publication.

Lundstedt et al. [2003] positively identified 117 compounds in the Husarviken soil and Table I highlights the degradation results for the 16 US EPA priority PAHs and selected PACs. Concentrations of priority PAHs ranged from 2.4 to 420 μg/g dry soil, and the chemical composition is similar to that noted for soils from other gasworks sites [Wilson and Jones, 1993; Kilbane, 1998; Eriksson et al., 2000; Saponaro et al., 2002], which typically contain a variety of high and low molecular weight PAHs and O- and S-heterocyclic compounds [Haeseler et al., 1999]. Oxy-PAHs, such as ketones, were also found in the Husarviken soil. These compounds have also been detected in other soils from former gasworks sites [Brooks et al., 1998; Eriksson et al., 2000; Saponaro et al., 2002].

**TABLE I:** Summary of Changes Observed in Polycyclic Aromatic Hydrocarbon and Polycyclic Aromatic Compound Levels Throughout the Bioremediation Treatment

		Fraction left in soil after degradation (%)			
Compound	Concentration in soil (μg/g dry soil)	Day 3	Day 7	Day 24	Day 29
Naphthalene	17	95	83	76	70
Acenaphthylene	29	78	74	81	73
Acenaphthene	2.4	22	14	0.3	0.3
Fluorene	44	27	19	4.1	2.6
Phenanthrene	330	34	18	8.7	6.4
Anthracene	70	63	52	24	14
Fluoranthene	420	93	84	47	37
Pyrene	290	101	94	65	50
Benz [*a*] anthracene	190	101	101	62	49
Chrysene	180	102	98	70	52
Benzo[*b*]fluoranthene	160	102	93	103	86
Benzo[*k*]fluoranthene	130	104	102	103	89
Benzo[*a*]pyrene	120	102	97	104	91
Indeno[1,2,3-*cd*]pyrene	100	101	94	105	101
Dibenz[*a,h*]anthracene	28	102	95	104	93
Benzo[*ghi*]perylene	84	101	94	106	102
9-Fluorenone	26	56	37	24	21
4-Oxapyrene-5-one		112	107	149	156
Benzofluorenones (3 peaks)		101	101	80	64
Benzothiophene	27	104	75	53	51
Carbazole	32	46	29	22	18
Benzo[*b*]naphtho[2,1 -d]thiophene		102	100	61	47

The results are normalized for the raw data obtained for anthracene; day 3: 82%, day 7: 100%, day 24: 79%, day 29: 107%. % degradation was determined by comparing peak areas to available reference standards; for complete data set see Lundstedt et al., 2003.

The general trends in PAH biodegradation observed by Lundstedt et al. were consistent with those observed in other studies [Cerniglia, 1992; Wilson and Jones, 1993; Eriksson et al., 2000]. The concentrations of most compounds decreased over the duration of the bioremediation treatment and, as expected, low molecular weight PAHs and heterocyclic compounds degraded more rapidly than high molecular weight PAHs. Also, alkyl-PAHs appeared to be more recalcitrant than the corresponding unsubsti-tuted PAHs.

The Lundstedt et al. work highlighted an increase in the concentration of oxy-PAHs over the course of the treatment, and noted that this outcome may contribute to the toxicological hazard of the treated soil. hi the Husarviken soil, two oxy-PAHs, 1-acenaphthenone and 4-oxa-pyrene-5-one (5H-phenanthro[4,5-*bcd*]pyranone), were specifically highlighted, and found to increase by 30 and 60%, respectively, over the course of the bioremediation. The concentrations of some oxy-PAHs, such as benz [*a*] anthracene-7,12-dione and 9-fluorenone, did not decrease as rapidly as their parent PAHs (i.e., benz [*a*] anthracene and fluorene) [Lundstedt et al., 2003]. This was unexpected, especially because oxy-PAHs have previously been found to be more bioavailable than their parent compounds [Matscheko et al., 2002], and are therefore expected to degrade more rapidly.

### Mutagenicity of Soil Fractions During Bioremediation

**Fig. 1:**
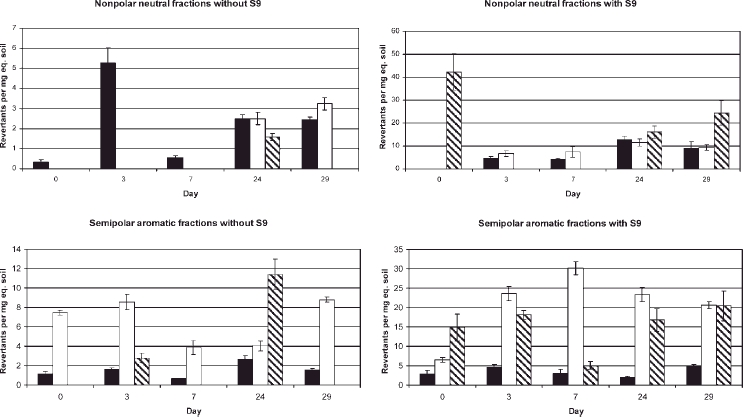
Direct-acting (i.e., −S9) and indirect-acting (i.e., +S9) mutagenic potencies of the nonpolar neutral and semipolar aromatic soil fractions measured during day 0, 3, 7, 24, and 29 of the bioremediation treatment in *Salmonella* strains TA98 (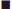), YG1041 (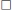), and YG1042 (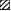). Note that TA100 did not detect any mutagenic activity in any samples on any days.

This study employed the Salmonella reverse mutation assay to assess the mutagenicity of nonpolar neutral and semipolar aromatic fractions of dried slurry extracts from day 0, 3, 7, 24, and 29 of the bioremediation treatment (i.e., same as the samples analyzed by Lundstedt et al., [2003]). Figure 1 summarizes the observed changes in mutagenic potencies throughout the course of the bioremediation. The combined values for the nonpolar neutral and semipolar aromatic fractions on TA98 indicate that the total mutagenic activity varies from 1.2 to 6.9 rever-tants per mg dry soil without S9 and 2.8 to 15 revertants per mg dry soil with S9. These values are consistent with heavily contaminated soils from industrial locations [White and Claxton, 2004].

### Nonpolar Neutral Compounds

The mutagenic activities of the nonpolar neutral fractions without S9 were relatively low compared to those of the semipolar aromatic fractions. Moreover, the mutagenicity of the nonpolar neutral fractions was highly variable over the course of the bioremediation. Mutagenic potencies ranged from 0.3 to 5.3 revertants per mg dry soil without S9. This low, yet measurable, level of mutagenic activity indicates the presence of direct-acting substances that may include PAH transformation products that elute in this fraction. In general, the mutagenic activity of the nonpolar neutral fractions was enhanced with the addition of S9, and the mutagenic potency values with S9 ranged from 4.2 to 43 revertants per mg dry soil (Fig. 1). This increase in mutagenicity upon the addition of S9 was expected because this fraction contains PAHs that are known to be indirect-acting mutagens that require metabolic transformation to electrophilic intermediates to react with DNA. Cytochrome P450 isozymes (e.g., Cyp1A1 and 1A2) present in the S9 fraction of rat liver transform PAHs into reactive metabolites that form DNA adducts, and contribute to the formation of stable mutations detected by the Salmonella mutation assay [Xue and Warshawsky, 2005].

The results for the nonpolar neutral fractions indicate that positive responses are more frequently observed with the *Salmonella* strains that detect frameshift mutations (i.e., TA98 and YG1041), in comparison with those that detect base-pair substitutions (i.e., TA100 and YG1042). Moreover, the highest levels of mutagenic activity without S9 were detected with these frameshift strains. In fact, TA100 did not detect any significant levels of mutagenic activity, with or without metabolic activation, in any sample. This was unexpected because previous studies have demonstrated strong base-pair substitution activity for PAHs and related compounds [McCann et al., 1975]. However, it should be noted that *Salmonella* strain YG1042 detected high mutagenic activities in the nonpolar neutral fractions with the addition of S9. The relative change in base-pair substitution activity between YG1042 and TA100 may suggest the presence of aromatic amines in this fraction; these compounds require metabolic activation by S9 and O-acetyltransferase to induce a mutagenic response. However, their presence in this fraction would be surprising, because validation of the fractiona-tion procedure by Lundstedt et al. has demonstrated that aromatic amines elute in the semipolar aromatic fraction [Lundstedt et al., 2003]. Alternatively, the S-heterocyclic compounds present in the nonpolar neutral fractions of the bioslurry extracts (e.g., benzothiophene and diben-zo[*b*]naphtha[2,1-*d*]thiophene) may be inducing a larger response on the metabolically enhanced *Salmonella* strain. Limited information is currently available about the pathways involved in metabolism and activation of mutagenic S-heterocyclic compounds and it is not known if the mutagenicity of these compounds can be modulated by Cnr and/or OAT.

Overall, a general trend toward increasing mutagenic activity in the nonpolar neutral fractions was observed as the treatment progressed, and this was particularly apparent with the metabolically enhanced strains (i.e., YG1041 and YG1042). This increase in mutagenicity was surprising because many of the compounds in this fraction decreased to 10% of their initial concentration over the course of the remediation [Lundstedt et al., 2003]. Moreover, it is commonly assumed that decreases in the concentration of priority substances are correlated with decreases in toxicological activity. Some studies have also shown that the genotoxicity of PAH-contaminated soils decreases during bioremediation [Aprill et al., 1990; Alexander et al., 2002], and as a result of soil aging [Alexander and Alexander, 1999]. However, others have shown substantial increases in genotoxicity following remediation [Hughes et al., 1998], or initial increases in mutagenicity followed by decreases during extended periods of remediation [Barbee et al., 1992, 1996]. The latter results as well as other published works that examined temporal changes in the mutagenic activity of contaminated soils undergoing remediation [e.g., Brown et al., 1986; Aprill et al., 1990; Donnelly et al., 1990] suggest that longer periods of remediation may be required to significantly reduce the mutagenic hazard of a contaminated soil. Thus, it seems clear that no single trend will be applicable to all contaminated sites and all bioremediation methods.

Interestingly, for the nonpolar fractions, some strains detected peaks of mutagenic activity at several time-points during the treatment. For example, TA98 detected maximal mutagenic activity without S9 on Day 3, and maximal S9-activated mutagenic activity on Day 24. The somewhat cyclical nature of the mutagenic activity may suggest the formation, and subsequent degradation, of mutagenic compounds; however, the identities of these muta-gens are unknown.

### Semipolar Aromatic Fractions

The semipolar aromatic fractions of the bioslurry samples generally exhibited higher mutagenic activity than their corresponding nonpolar neutral fractions, suggesting that the compounds present in these fractions (i.e., oxy-PAHs, nitro-PAHs, aromatic amines) are more abundant or more potent than those in the nonpolar fractions. Moreover, the addition of S9 almost always resulted in an increase in mutagenicity (Fig. 1). This result was not anticipated, since this fraction would be expected to contain oxy-PAHs and nitro-PAHs that are mainly direct-acting mutagens that induce frameshift mutations [Choudhury, 1982; Eide et al., 2002] and whose activity is frequently decreased with the addition of S9 [Choudhury, 1982; Rosenkranz and Mermelstein, 1983]. The elevated level of S9-mediated activity observed in this study was particularly noteworthy for the metabolically enhanced *Salmonella* strains (i.e., YG1041 and YG1042), and therefore may be due to the presence of oxygenated aromatic amines in the soil, rather than oxy-PAHs or nitroarenes. Aromatic amines require oxidation of the amino group to a hydroxylamine by CYP enzymes, whereas oxy-PAHs and nitroarenes are generally direct-acting mutagens that are active without S9. Aromatic amines, including N-heterocyclic compounds, were detected in the soil by Lundstedt et al. [2003] and some of these compounds are known to be potent mutagens [Tokiwa and Ohnishi, 1986; Aeschbacher and Turesky, 1991]. Alternatively, this increased activity could be due to the presence of oxygenated derivatives of S-heterocy-clic compounds, which would be expected to elute in this fraction, and that are in fact mutagenic. Kumar et al. [2004] demonstrated that the sulfoxide metabolite of benzo[*b*]naphtha[2,1-*d*]thiophene is mutagenic in TA100 with and without metabolic activation [Kumar et al., 2004], and a recent study by Swartz et al. demonstrates that some dihydrodiol and sulfone derivatives of benzo[c]phenanthrene, phenanthro[3,4-*b*]thiophene, and phenanthro[4,3-*b*]thiophene are mutagenic in TA98, TA100, and YG1041 [Swartz et al., in press].

Like the nonpolar fractions, the semipolar aromatic fractions also exhibited far greater activity in the frame-shift strains (i.e., TA98 and YG1041), as compared to the base-pair substitution strains (i.e., TA100 and YG1042), with no activity observed in TA100 at all. This observation was surprising since strong base-pair activity has previously been observed for PACs, including some oxy-PAHs [Moller et al., 1985] expected to be present in these bioslurry fractions.

The mutagenic potencies of the semipolar aromatic fractions also displayed considerable temporal variability, with peaks in activity observed at various points during the treatment. Although the treatment was terminated at 29 days, the results suggest a cyclic pattern of formation and degradation of mutagenic substances similar to that noted for the nonpolar neutral fractions. Moreover, the pattern of responses across strains and S9 conditions suggests the formation and degradation of specific types of substances at different points throughout the treatment. For example, without S9, the activity of the semipolar fraction in YG1042 drops significantly between days 24 and 29, while the activity in YG1041 increases. This suggests the degradation of some compounds that are frame-shift mutagens, and the formation of other compounds that induce base-pair substitutions. Other significant changes include a peak in activity in the absence of metabolic activation for YG1042 and TA98 on Day 24; this peak was not apparent with YG1041 (Fig. 1). In contrast, YG1041 direct-acting activity displayed a distinctly cyclic pattern with the maximum value observed at the end of the treatment (i.e., Day 29). The formation of a variety of PAH transformation products via biological processes during remediation has been documented. For example, two studies have noted the formation of hydroxylated, oxygenated, and methoxy derivatives during microbial and fungal degradation of PAHs [Wunder et al., 1997; Andersson et al., 2003]. In some instances the transformation products may be more persistent, bioavailable, or mutagenic than the parent compounds.

The mutagenicity of the bioslurry fractions cannot be fully explained by the corresponding chemical profiles presented in Lundstedt et al. [2003]. This is not unexpected and several authors have noted a distinct lack of correspondence between the results of toxicological analyses and matched chemical analyses [e.g., Brack et al., 2007]. The expected lack of correspondence confirms the presence of hitherto unknown mutagens that are likely being formed during the bioremediation treatment. For example, it is plausible that oxygenated aromatic amines are formed during the bioremediation process and are the causative agents of some of the increases in the mutage-nicity that were observed during the treatment. Formation and accumulation of some polar transformation products, such as oxy-PAHs, is of particular concern since such products are more water soluble, and consequently display higher environmental mobility, than their parent PAHs. Lundstedt et al. noted that the increased relative mobility of oxy-PAHs may significantly augment ecological hazard [Lundstedt et al., 2007]. Moreover, several oxy-PAHs are known to be toxic and mutagenic [Lundstedt et al., 2007]. We have assessed the mutagenic activity of one oxy-PAH highlighted by Lundstedt et al. (i.e., 4-oxapyrene-5-one); however, it failed to induce a positive mutagenic response in *Salmonella* strains TA98 and YG1041 (data not shown). Screening of other suspected agents and/or testing of oxy-PAHs and other PAH transformation products with different test systems, such as in vitro mammalian muta-genicity assays, may prove useful.

This study is one of very few that has simultaneously tracked changes in both mutagenic activity and chemical profile for a contaminated soil undergoing bioremediation. Similar studies include that of Morelli et al. [2001], who observed a similar pattern of increasing and decreasing *Salmonella* mutagenicity for sludge-amended soils contaminated with PAHs [Morelli et al., 2001]. Belkin et al. [1994] measured the genotoxicity of PAH-amended soil and PAH-contaminated soil with the Muta-tox assay during a 3-month biodegradation period [Belkin et al., 1994]. They found that direct-acting genotoxicity was actually enhanced following the degradation of high molecular weight PAHs. In another study, Donnelly and colleagues [2005] demonstrated an increase in the genotoxicity of a soil amended with benzo[a]pyrene during a 720-day bioremediation, despite the fact that less than 10% of the benzo[a]pyrene remained at the end of the treatment [Donnelly et al., 2005]. The authors attributed the increase to an accumulation of genotoxic metabolites, including oxy-PAHs. Alexander and Alexander [1999] reported a net decrease in mutagenicity for PAH-contaminated soil as the soil PAHs age. However, the reduction in mutagenic activity may be the result of reduced bioavailability of the PAHs or the PAH metabolites, rather than reduced mutagenic activity of the chemical constituents, in the aged soil [Alexander and Alexander, 1999].

## OVERALL CONCLUSIONS

In the absence of a more thorough, rigorous investigation that includes extended bioslurry treatment and a variety of treatment conditions, as well as simultaneous chemical and toxicological analyses, it is difficult to determine the underlying causes of the temporal changes in mutagenicity observed in this and other studies. Moreover, the ultimate risk of adverse health and environmental effects posed by soils undergoing bioremediation will also be determined by alterations in physical-chemical properties that control environmental fate, transport, and exposure. Future studies should employ extended bioremediation treatments, a variety of treatment conditions (e.g., nutrient augmentation), and continued examination of the chemical and toxicity profiles to determine the treatment duration required to reduce toxicity, hazard, and risk to background levels. It should be noted that reduction to background does not imply complete elimination of mutagenic activity, hazard, and risk. Several researchers [e.g., Jones and Peace, 1989; Donnelly et al., 1995; White and Claxton, 2004] have noted that natural background levels of mutagenic activity can be expected to be less than 0.1 revertants per mg dry soil. Moreover, Jones and Page [1989] note that levels above 1 revertant per mg should be cause for concern.

The results obtained in this study, as well as earlier work cited above, indicate that declines in the concentration of noteworthy soil contaminants (e.g., priority PAHs) do not necessarily correspond to declines in the genotoxic activity of a complex pollutant mixture such as that present in a contaminated soil. Although bioremediation is a viable option for the treatment of PAH contaminated soils, additional research will be required to determine if declines in both the level of PAHs and the level of mutagenic activity can be achieved in a reasonable time frame. The combined results of the Lundstedt et al. [2003] study and the current study show that bioremediation of PAH-contaminated soils is associated with the formation of specific PAH transformation products (e.g., 4-oxapyrene-5-one), as well as augmentation of mutagenic activity. At the present time, the identities of the putative mutagens in the treated soils are not known. Although some by-products of PAH and PAC metabolism are known mutagens, (e.g., dihydrodiol derivates of benzo[a]pyrene), and increases in the concentration of PAH transformation products were documented by Lundstedt et al. [2003], the mechanistic relationship between the changes in chemical composition and changes in mutagenic activity is unsubstantiated and poorly understood. Identification of the putative mutagens present at different treatment stages, and subsequent identification of the transformation pathways will require a considerable effort. Nevertheless, this type of research, which could employ bioassay-directed fractionation techniques such as those highlighted by Watanabe et al. [2008] to track (geno)toxic activity across selected chemical classes, can identify hitherto unknown (geno)toxic substances at contaminated sites, thereby improving the accuracy and utility of chemical-specific hazard assessments. Moreover, identification of the treatment conditions (e.g., duration, nutrient concentration) and microbial factors that control the mineralization of PAHs and PACs will enhance the reliability and utility of bioremediation. Routine monitoring of PAH degradation products and periodic testing for (geno)toxic activity could ultimately be employed to evaluate remediation options and treatment efficacy.
